# Investigating the Structural, Thermal, Electric, Dielectric, and EMI Shielding Properties of Porous Thermoplastic Polyurethane Reinforced with Carbon Fiber/Magnetite Fillers

**DOI:** 10.3390/polym18010019

**Published:** 2025-12-21

**Authors:** Hülya Kaftelen Odabaşı, Ümmühan Kaya, Akın Odabaşı, Selçuk Helhel, Fernando Ruiz-Perez, Felipe Caballero-Briones

**Affiliations:** 1 Department of Aircraft Maintenance and Repair, School of Civil Aviation, Fırat University, 23200 Elazığ, Turkey; 2 Graduate School of Natural and Applied Science, Aerospace Science & Technologies, Fırat University, 23200 Elazığ, Turkey; ummhan.kayaa@gmail.com; 3 Department of Metallurgical and Materials Engineering, Engineering Faculty, Fırat University, 23200 Elazığ, Turkey; odabasia@firat.edu.tr; 4 Department of Electrical & Electronics Engineering, Engineering Faculty, Akdeniz University, 07058 Antalya, Turkey; selcukhelhel@akdeniz.edu.tr; 5 Instituto Politécnico Nacional, Materiales y Tecnologías para Energía, Salud y Medio Ambiente (GESMAT), CICATA Altamira, Altamira 89600, Mexico; ruizpfernando@hotmail.com (F.R.-P.); fcaballero@ipn.mx (F.C.-B.)

**Keywords:** electromagnetic shielding, thermoplastic polyurethane composites, carbon fiber, Fe_3_O_4_ particles, porous structure

## Abstract

In this study, Fe_3_O_4_-chopped carbon fiber (CF) fillers with varying CF:Fe_3_O_4_ weight ratios (1:0.5, 1:0.75, and 1:1) were fabricated using the wet chemical reduction method. Different weight percentages (1, 3, 7 wt.%) of the CF/Fe_3_O_4_ fillers were used to fabricate lightweight, flexible, and porous thermoplastic polyurethane (p-TPU) composites for electromagnetic interference (EMI) shielding applications. Due to its poor electrical and magnetic properties, the TPU matrix alone exhibited negligible shielding effectiveness. The electromagnetic interference (EMI) performance of TPU composites is greatly affected by the amount of filler materials, the CF/Fe_3_O_4_ ratio, and the porous structure, which in turn influence the interfacial interactions between filler and p-TPU matrix. Effective electromagnetic attenuation is achieved by conductive CF network, interfacial polarization at CF/Fe_3_O_4_/TPU interfaces, and multiple internal reflections promoted by microstructural heterogeneity and porosity. A maximum EMI shielding effectiveness (SE_T_) of 22.28 dB was achieved for a CF/Fe_3_O_4_/p-TPU composite with a filler load of 7 wt.%, a CF:Fe_3_O_4_ ratio of 1:1, and a porosity of 15%.

## 1. Introduction

The electromagnetic (EM) waves emitted by the increasing number of electronic devices can interfere with the functioning of other electronics and may pose risks to human health [1,2,3]. To meet this challenge, there is a growing need for the development of high-performance EMI shielding materials. Despite their effectiveness, conventional metal-based shielding materials are limited by their high density, lack of flexibility, and susceptibility to corrosion [4,5]. In contrast, polymer-based composites have emerged as promising EMI shielding materials, offering lightweight, flexibility, broad absorption bandwidth, and high processability [6,7,8]. Among EMI shielding materials, polymer composites with carbon fillers are frequently preferred due to their conductivity, high strength, and corrosion resistance; additionally, carbon-based composites offer the advantage of increasing electrical conductivity with minimal filler content, thereby effectively reducing the overall product cost [9,10,11,12,13,14].

Thermoplastic polyurethane (TPU) offers high elasticity, impact resistance, and abrasion resistance, making it widely used as a matrix material in EMI shielding applications. Furthermore, TPU can easily integrate with various fillers to modify the reflection and absorption of EM waves when combined with additives like carbon fiber and magnetite (Fe_3_O_4_) [14,15,16,17,18,19]. Carbon fiber is a lightweight material with excellent electrical conductivity, which allows for effective reflection of EM waves, while its mechanical strength and thermal stability make it an ideal candidate for EMI shielding materials [20,21]. Magnetite (Fe_3_O_4_), on the other hand, is known for its high magnetic permeability and ability to form magnetic dipoles, which facilitate the absorption of EM waves, thus increasing the shielding effectiveness [15,16]. For example, Zhang et al. [16] demonstrated that by incorporating Fe_3_O_4_-coated carbon nano fibers into a TPU matrix, up to 97% of EM waves were absorbed.

Porous structures have been proposed for achieving multiple scattering of EM waves, resulting in increased energy dissipation and enhanced absorption within the shielding material [21]. A study by Gao et al. [22] showed that porous composites reinforced with carbon-based fillers exhibited high-performance EMI shielding. Similarly, in a study by Theertharaman et al. [23], porous composites containing Fe_3_O_4_ and carbon black achieved 99.99% reflection in the X-band frequency range, with a shielding effectiveness of 66.95 dB.

Despite the number of publications on TPU composites and the combined use of carbon-based and magnetic fillers, few extensive reports include the characterization of the dielectric and magnetic properties of the materials with a study of the materials properties, including the interactions between the filler and the matrix. Therefore, in this study, EMI shielding composites were prepared using a porous TPU matrix filled with different amount of previously activated, chopped carbon fibers decorated with Fe_3_O_4_ particles with varying CF:Fe_3_O_4_ ratios. The effect of CF:Fe_3_O_4_ ratios, the filler amount, the interaction between the filler and the matrix, and the porosity of the composites on the EMI shielding performance of the p-TPU-based composites was investigated in the X-band frequency range. The novelty of this study lies in establishing systematic correlations between the microstructural properties of composites, porosity, electromagnetic parameters (dielectric permittivity, magnetic permeability, impedance matching, skin depth estimation, shielding components), conductivity, and absorption-dominated EMI shielding behavior.

## 2. Experimental

### 2.1. Materials

In this study, commercial-grade Ravathane AG190-grade polyester-based TPU granules (shore hardness: 97, density 1.21 g/cm^3^, Ravago Co. (Istanbul, Türkiye)) used as the matrix material were kindly donated by Ravago Co. (Istanbul, Türkiye). The chopped carbon fiber (6 mm diameter) with a density of 1.81 g/cm^3^ was purchased from Dost Kimya Co. (Istanbul, Türkiye). For Fe_3_O_4_ synthesis, iron (III) chloride hexahydrate (FeCl_3_·6H_2_O) (99.0% purity) was used as the precursor, and sodium borohydride (NaBH_4_, 98% purity) as the reducing agent. Nitric acid (HNO_3_) was used for surface treatment of carbon fibers, oleylamine (technical grade 70%) to improve dispersion, and acetone (CH_3_COCH_3_) as the solvent, respectively.

### 2.2. Synthesis of CF/Fe_3_O_4_ Filler Materials

First, carbon fibers were surface activated as follows: 1 g of CF was treated at 500 °C in an argon (Ar) atmosphere for one hour. Then, CF was stirred in 60 mL of concentrated nitric acid solution at 65 °C for 2 h. After treatment, the CF was centrifuged, rinsed with deionized water, neutralized to pH 7, and then dried at 80 °C for 12 h [24].

CF/Fe_3_O_4_ fillers were prepared by the synthesis of Fe_3_O_4_ nanoparticles via the wet chemical reduction method [25]. A certain amount of activated CF was introduced into an aqueous solution of 0.2 M FeCl_3_·6H_2_O containing 80 mL of distilled water. This mixture was stirred in an oil bath at 60 °C for 5 h. For reduction, 20 mL of a NaBH_4_ solution was slowly added. Oleylamine was added after the reaction to enhance stability and improve dispersion of CF, with further stirring for 30 min. The final CF/Fe_3_O_4_ filler was obtained after washing with 100 mL of deionized water three times to remove NaCl by-product, and later drying the material at 80 °C for 24 h [25]. For the magnetic and morphology characterization, a batch of Fe_3_O_4_ nanoparticles was synthesized using the same procedure.

### 2.3. Preparation of CF/Fe_3_O_4_/p-TPU Composites

The hybrid CF/Fe_3_O_4_/p-TPU composites were prepared by the solution casting method. First, 20 g of TPU granules were dried under vacuum at 75 °C for 24 h. The dried granules were dissolved in an acetone solution at a 4:1 ratio and stirred using a magnetic stirrer at 400 rpm for 20 h in a sealed borosilicate vessel maintained at 30.0 ± 0.5 °C to ensure complete dissolution while minimizing solvent loss. CF/Fe_3_O_4_ filler was added to this solution in the desired ratios, then pre-mixed in 40 mL of acetone, stirred for 30 min, and dispersed using an ultrasonic bath for 10 min. The resulting CF/Fe_3_O_4_/TPU solution was stirred on a magnetic stirrer for 4 h, and a homogeneous solution was visually inspected for residual particulates prior to casting. The CF/Fe_3_O_4_/TPU solution was cast into a Teflon-coated Petri dish, forming a uniform wet layer with a target thickness of 3.5 ± 0.05 mm, measured at five equidistant positions using a digital micrometer (measurement uncertainty ±5 μm).

**Figure 1:**
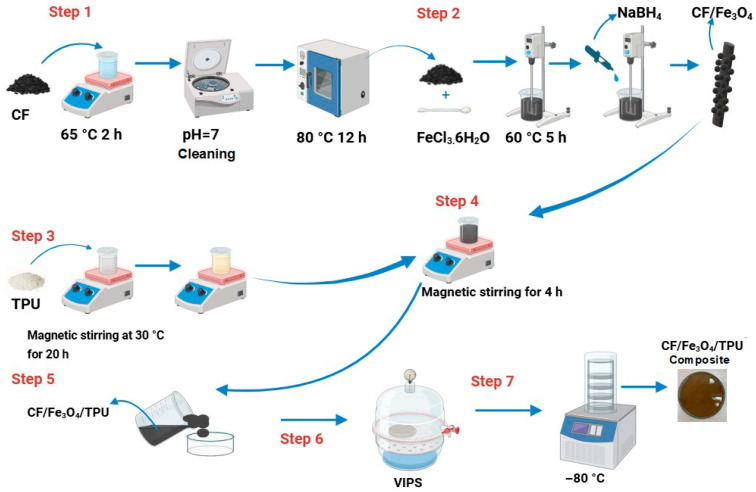
Schematic illustration of the preparation process of CF/Fe_3_O_4_/p-TPU composites. The schematic is not drawn to scale and does not represent the actual dimensions of the components.

Porous polyurethane (p-TPU) composites were prepared using acetone–water vapor exchange (vapor-induced phase separation, VIP). The cast film was immediately transferred to a room-temperature desiccator to initiate VIPS. Distilled water at 25.0 ± 0.2 °C was placed in the desiccator reservoir to generate a controlled water-vapor environment. The polyurethane (p-TPU) composites were exposed to this vapor atmosphere for 24 h, during which temperature was continuously monitored using a calibrated data logger (uncertainty ±0.2 °C), and exposure time was recorded to an accuracy of ±0.1 s. Relative humidity inside the desiccator remained stable within the sensor’s uncertainty of ±2% RH. Following VIPS, the phase-separated TPU sample was frozen at −80 °C for a minimum of 2 h to preserve the internal morphology before drying. Lyophilization was performed using a freeze-dryer operating under a vacuum of ≤0.05 mbar. The drying program consisted of a primary drying stage at −40 °C for 12 h, followed by a controlled shelf-temperature increase to 20 °C over 6 h to complete sublimation of both acetone and water. The dried samples were stored in a desiccator until further use. All experiments were conducted in triplicate (*n* = 3) using independently prepared casting solutions. Process parameters (temperature, exposure time, thickness, and RH) were monitored and reported with their respective measurement uncertainties. The composites were cut into appropriate dimensions (10 mm × 20 mm × 3.5 mm) for EMI shielding measurements, with a neat p-TPU sample prepared using the same method for comparison. The preparation process of CF/Fe_3_O_4_/p-TPU composites is schematically illustrated in Figure 1.

In this study, two sets of hybrid CF/Fe_3_O_4_/pTPU composites were prepared. In the first set, the p-TPU matrix contains 1 wt.% CF/Fe_3_O_4_ with different CF:Fe_3_O_4_ weight ratios of 1:0.5, 1:0.75, and 1:1 and they were named as TCP105, TCP107, and TCP110 composites, respectively. In the second set, the p-TPU matrix containing 3 and 7 wt.% CF/Fe_3_O_4_ filler with different weight ratios of 1:0.75 and 1:1, and the samples are named as follows: TCP 307, TCP310, TCP707, and TCP710.

Samples are summarized in Table 1. The first number in sample names indicates that the TPU composites the wt.% of the CF/Fe_3_O_4_ filler (1, 3, 7), and the second figure indicates the CF:Fe_3_O_4_ weight ratio (0.5, 0.75, 1). For example, TCP-105 represents the p-TPU composite material containing 1 wt.% of the CF/Fe_3_O_4_ filler with a CF:Fe_3_O_4_ ratio of 1:0.5. In Table 1, the average density and standard deviation, calculated using Equation (1) and the porosity, calculated with Equation (2), are also displayed. The trends and the influence of the density and porosity on the EMI shielding efficiency are discussed later.

### 2.4. Characterization

The X-ray diffractogram of the Fe_3_O_4_ nanoparticles was collected in a Bruker D8 Advanced diffractometer (CuKα radiation at λ = 1.5418 Å) at 30 mA and 40 kV, in Bragg–Brentano geometry, with a 0.02° step. The Fe_3_O_4_ nanoparticles were observed in a transmission electron microscope (TEM) JEOL-1010 equipped with an ORIUS camera (Gatan) at 80 kV. The specific magnetization of the Fe_3_O_4_ nanoparticles and the TCP710 composite was measured by vibrating sample magnetometry (VSM) using a Quantum PPMS EverCool-I 9T system. The curves were obtained at 300 K, scanning the magnetic field between ±30 kOe. Fourier Transform Infrared Spectroscopy (FTIR) of the activated CF fibers was performed using a Shimadzu spectrometer in the transmission geometry from 4000 to 400 cm^−1^. The Fe_3_O_4_ nanoparticle distribution in the carbon fibers was assessed via Scanning Electron Microscopy (SEM, Zeiss Supra 55 FESEM (New York, NY, USA)), while the elemental carbon, iron, and oxygen distribution in the CF/Fe_3_O_4_ filler was verified using Energy Dispersive Spectroscopy (Bruker EDS). The fracture surfaces of the CF/Fe_3_O_4_/p-TPU composites were examined using SEM using an excitation voltage of 20 kV. The CF/Fe_3_O_4_/p-TPU composites were fractured in liquid nitrogen for SEM examination to observe the porous structure and the distribution of the CF/Fe_3_O_4_ filler within the p-TPU matrix. From the SEM images, the pore sizes were measured using the open-source ImageJ 1.38 software [26] as described in the procedure depicted in the Supplementary Information section. Thermogravimetric analysis was conducted under nitrogen flow from room temperature to 600 °C at a heating rate of 10 °C min^−1^ in a Shimadzu (DTG-60) TG/DTA Analyzer. The densities of neat p-TPU and the CF/Fe_3_O_4_/p-TPU composites were measured using the water displacement method according to ASTM D792, using a 25 mL specific pycnometer. The density of the composite samples was calculated according to Equation (1):

(1) $$\rho_{exp}=\frac{m_{c}}{m_{c}+m_{i}-m_{l}}\rho_{H_{2}O}$$

where $m_{c}$ is the weight of the sample, $m_{i}$ is the initial weight of the pycnometer filled with distilled water, and $m_{l}$ is the weight of the composite sample immersed in a pycnometer filled with distilled water. $\rho_{H_{2}O}$ in the formula shows the density of pure water. Sample weighing was carried out using a 4-digit precision balance. Composite samples were immersed in a pycnometer filled with water, allowed to equilibrate for half an hour, and weighed. The procedure was repeated 5 times per sample. The density data of the composites and the standard deviation are shown in Table 1.

The theoretical density ($\rho_{th}$), was calculated from Equation (2) [8]:

(2) $$\rho_{th}=(1-x)\rho_{TPU}+x\rho_{CF/{Fe}_{3}O_{4}}$$

where x is the volume fraction of fillers. Here, the theoretical density values of the $\rho_{CF/{Fe}_{3}O_{4}}$ fillers, were taken as 2.310 g/cm^3^, 2.513 g/cm^3^, 2.68 g/cm^3^, 2.378 g/cm^3^, 2.679 g/cm^3^, 2.507 g/cm^3^, 2.685 g/cm^3^ for TCP105, TCP107, TCP110, TCP307, TCP310, TCP707, and TCP710, respectively. From these values, the ASTM D2734 approach was used to determine the void content, i.e., the porosity of the composites using Equation (3), the results of which are also displayed in Table 1 [27]:

(3) $$Porosity\;\left(\%\right)=\frac{\rho_{th}-\rho_{exp}}{\rho_{th}}$$

The electromagnetic properties of the composites were assessed by measuring dielectric and magnetic permeability values (ε′ and ε″, μ′ and μ″) using an Agilent N5247A Vector Network Analyzer (VNA). The electromagnetic properties (ε′, ε″, μ′, and μ″) of the composites were retrieved from the measured S_11_ and S_21_ using the robust Nicolson–Ross–Weir (NRW) method, the standard analytical approach for waveguide-based material characterization. To ensure that the derived permittivity and permeability values (ε_r_, µ_r_) were physically smooth and free from mathematical artifacts, time-domain gating was applied to the measured S-parameters. The purpose of this technique was to successfully isolate the primary reflection and transmission pulses from unwanted periodic reverberations (ringing) originating within the measurement setup. The optimal gating time was determined to be tgate = −0.1 ns, as this was the minimum negative temporal shift required to completely eliminate the oscillations in both the ε’ and μ’ spectra. More aggressive (more negative) t_gate_ settings were avoided, as they would risk truncating the sample’s true primary physical reflection data, leading to the calculation of inaccurate material parameters.

An Agilent N5247A Vector Network Analyzer (VNA) was used as the base instrument to measure the scattering parameters (S_11_ and S_21_) in the proposed frequency range, which is X-band. All measurements were performed in WR-90 waveguides using an HP VNA with full SOLT (open-short-load-thru) calibration and Huber and Suhner low-loss cables working up to 28.5 GHz, ensuring accurate reference-plane definition. All samples were machined to a thickness of 3 ± 0.1 mm, with dimensions of 10 mm × 22 mm, thereby filling the waveguide cross-section to provide proper boundary conditions. The measurement setup/procedure and parameter retrieval methods have already been proven [28,29,30,31]. The porous TPU composites were cast in molds and sanded to achieve uniform thickness; dimensional tolerances were verified with a digital micrometer. Each sample was measured three times, removing and reinserting between runs to evaluate repeatability. The laboratory performs antenna and RADOME characterization up to 28.5 GHz, ensuring stable environmental conditions (temperature: 23 ± 1 °C) and instrumentation conditions. In the NRW formulation, the complex reflection (Γ) and transmission (T) coefficients are computed from S-parameters. The AC electrical conductivity of the composites was determined using Equation (4):

(4) $$\sigma_{ac}=2\pi f\varepsilon_{0}\varepsilon^{\prime \prime }$$

where ƒ is the frequency in Hz, $\varepsilon_{0}$ is the permittivity of vacuum (8.854 × 10^−12^), and ε″ is the imaginary part of the permittivity. The ε″ values of the composite samples were measured using vector network analysis with the coaxial line method for plane wave conditions.

The scattering parameters (S_11_ and S_21_) in the frequency range of 8.2–12.4 GHz were collected to calculate EMI SE (SE_total_), reflection loss (SE_R_), and absorption loss (SE_A_) using Equations (5)–(10):

(5) $$R={\left|S_{11}\right|}^{2}$$

(6) $${T=\left|S_{21}\right|}^{2}$$

(7) A=1−R−T

(8) $${SE}_{R}=-10lg(1-R)$$

(9) $${SE}_{A}=-10lg\left\lfloor T/(1-R)\right\rfloor$$

(10) $${SE}_{total}={SE}_{R}+{SE}_{A}+{SE}_{M}$$

where ${SE}_{M}$ represents the microwave multiple internal reflections, and can be negligible when ${SE}_{total}$ is higher than 10 dB [32]. Dielectric and magnetic permeability values were used to analyze the effects of filler content and the porous structure on EMI shielding.

Finally, intrinsic impedance and skin depth (δ) were calculated using the following equations:

(11) $$Z=\;\sqrt{\frac{j\omega \mu }{\sigma +j\omega \varepsilon }}$$

(12) $$\delta =\sqrt{\frac{2}{\omega \mu_{0}\mu_{r}\sigma }\;}$$

where ω is the angular frequency (2πf), µ is the magnetic permeability, ε is the electric permittivity, σ is the conductivity, µ_0_ is the permeability of free space (1.256 $\times \;10^{-6}$ Hm^−1^), and *j* is the imaginary unit.

The DC electrical conductivity of TPU composite samples was determined using a four-point probe system (Entek Elektronic Co., Ltd., Istanbul, Turkey). Electrical conductivity measurements were conducted on three samples with the same composition at five different locations. Conductivity tests were performed on samples with a thickness of 3 mm at room temperature.

The resistivity ($\rho_{0}$ Ω cm) and conductivity (σ, Scm^−1^) values were calculated using the following equations [33]:

(13) $$\rho_{0}=\left(\frac{V}{I}\right)\times 2\pi s$$

(14) σ=1ρ0

where V is the potential difference between the probes, I is the current through the outer pair of probes, and s is the spacing between the probes. In this measurement, the sample thicknesses are t >> s, probe spacing (1 mm).

## 3. Results and Discussion

### 3.1. Characterization of the Filler Precursors

**Figure 2:**
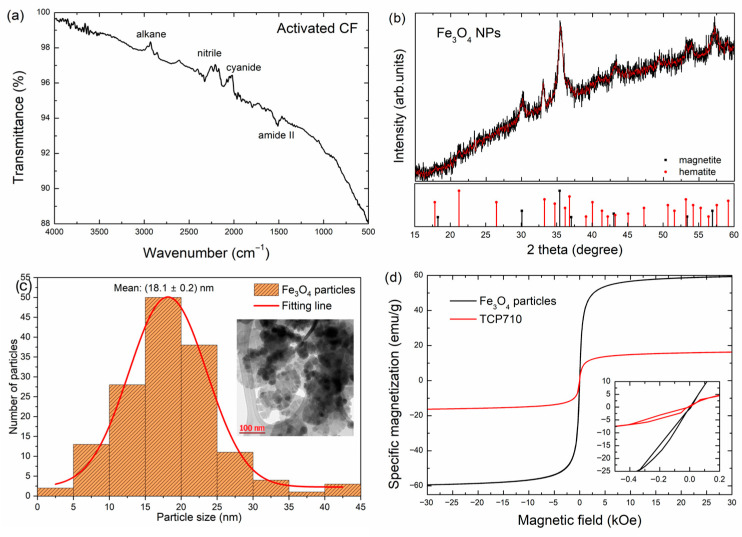
(**a**) FTIR spectrum of the activated CF; (**b**) X-ray diffractogram of Fe_3_O_4_ NPs after washing; (**c**) Particle size distribution and TEM micrograph of Fe_3_O_4_ NPs; (**d**) Magnetization hysteresis curves of Fe_3_O_4_ NPs and of the TCP710 composite.

To assess the characteristics of the materials properties, Figure 2 presents (a) an FTIR spectra of the activated CF; (b) an X-ray diffractogram (XRD) of the Fe_3_O_4_ nanoparticles after the washing procedures; (c) a TEM micrograph and the particle size distribution of the Fe_3_O_4_ nanoparticles, and (d) the hysteresis curves of the Fe_3_O_4_ nanoparticles and the TCP710 sample.

The FTIR of the activated CF in Figure 2a shows bands related to alkane (CH_3_/CH_2_) moieties at about 2850–2950 cm^−1^, nitrile (2170–2250 cm^−1^) and cyanide (2000–2056 cm^−1^), as well as a band at ca. 1500 cm^−1^ related to the amide II group. The presence of nitrogen-related groups is directly attributed to the activation with HNO_3_ and may direct the magnetite nucleation [34]. The X-ray diffractogram of the washed nanoparticles shows peaks at 2θ = 30.1°, 35.2°, 43°, and 56.6°, associated with the (111), (311), (511), (440), and (533) planes of magnetite (Fe_3_O_4_, PDF 19-629), with the space group Fd-3m [28]. Also, peaks 21.1°, 32.9°, 36.4°, and 56.9°, related to the (110), (130), (111), and (221) planes of hematite (Fe_2_O_3_, PDF 33–664) were observed, which are possibly due to oxidation during manipulation. In Figure 2c, a monodisperse size distribution of the nanoparticles centered at 18 ± 2 nm was calculated from the TEM image shown in the inset, where quasispheroidal particles can be observed. Finally, Figure 2d presents the magnetic hysteresis curves of the Fe_3_O_4_ nanoparticles and that of the TCP710 composite, i.e., with the highest filler loading (7% wt.) and the highest CF:Fe_3_O_4_ ratio (1:1). The magnetic hysteresis of the nanoparticles is typical of soft magnetic materials [35], with a low coercive field (ca. 0.05 kOe) and a high specific magnetization, ca. 60 emu/g. In contrast, the specific magnetization lowers to ca. 18 emu/g for the p-TPU-based composite; this behavior is like that reported for CoFe_2_O_4_/TPU composites with filler contents around 7.5 wt.%, and it is associated with good filler dispersion in the matrix [36].

### 3.2. Morphology of the CF/Fe_3_O_4_ Filler

**Figure 3:**
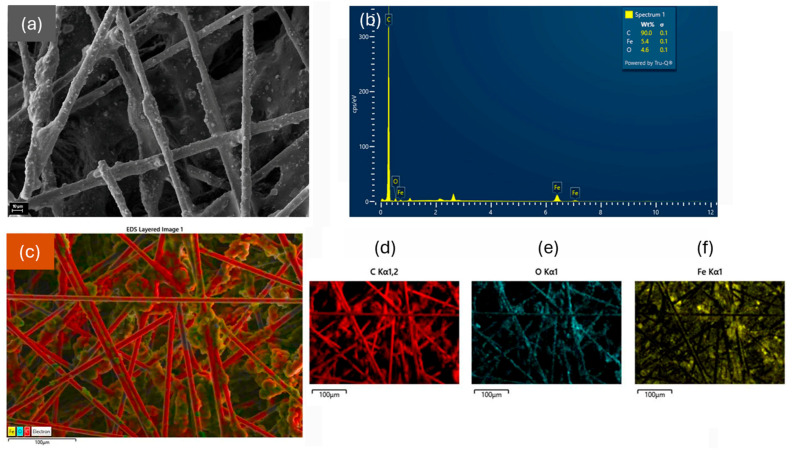
(**a**) SEM micrograph of the filler with a CF:Fe_3_O_4_ ratio of 1:0.5, (**b**) EDS spectrum with elemental composition, (**c**) superimposed image from the EDS maps of C (**d**), O (**e**), and Fe (**f**).

Figure 3 presents the SEM/EDS analysis performed on the filler CF/Fe_3_O_4_ with a ratio of 1:0.5, to assess the morphology, composition, and magnetite distribution onto the carbon fibers. Figure 3a presents the micrograph of the sample, where the fibers covered with particles and agglomerates are noticed. Figure 3b provides an energy dispersive spectra (EDS) where the signals are C (90 wt.%), Fe (5.4 wt.%), and O (4.6 wt.%). Figure 3d–f shows the elemental mapping of the CF/Fe_3_O_4_ (1:0.5) filler. A superimposed element distribution map is shown in Figure 3c, where the presence of nanoparticles is observed along the entire fibers, with regions of agglomerated particles. The concentration of Fe_3_O_4_ particles along the entire carbon fibers indicates the interaction between the particles and the fibers, due to the previous activation of the CFs [35,36]. In summary, the XRD, VSM, and SEM/EDS data highlight the presence of magnetic Fe_3_O_4_ nanoparticles interacting with the activated carbon fibers, leading to soft magnetic behavior in the composites, which would influence the EMI shielding properties of the material as discussed later.

### 3.3. Morphological Analysis of CF/Fe_3_O_4_/p-TPU Composite

**Figure 4:**
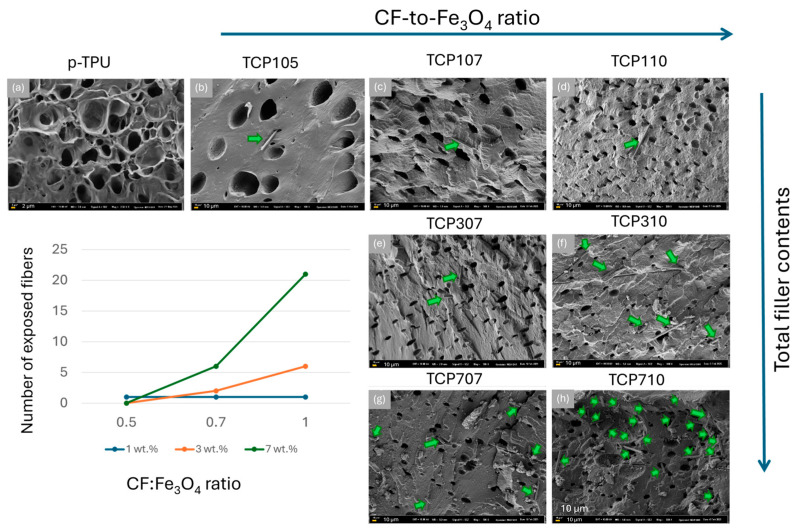
SEM micrographs of (**a**) p-TPU, (**b**) TCP105, (**c**) TCP107, (**d**) TCP110, (**e**) TCP307, (**f**) TCP310, (**g**) TCP707, and (**h**) TCP710. Inset shows the number of exposed fibers as determined from the micrographs (green arrows) with respect to the CF:Fe_3_O_4_ ratio for the three-filler contents (wt.%). Image scale bars are 10 µm, except for p-TPU, which is 2 µm.

SEM micrographs in Figure 4 display the cryo-fracture surfaces of porous TPU (p-TPU) and its composites with different ratios of CF:Fe_3_O_4_ and filler contents, as described in Table 1. Figure S4 in the Supplementary Information section shows the pore size distributions obtained from the SEM images, while Table 2 presents the mean porosity values, the standard deviation, as well as the maximum pore sizes achieved at each sample. The pores promote EMI shielding by facilitating multiple scattering of electromagnetic waves at the composite volume.

Figure 4a shows that neat p-TPU, which attracts attention with its asymmetric pore structure, with both open and closed cell pore structures due to the VIP process; this sample has a maximum pore size of 11 μm and an average pore size of 2.7 ± 0.9 μm. Figure 4b–h shows the SEM images of the prepared composites, where the pore structure and distribution of TPU composites are observed to change with the filler contents and with the CF:Fe_3_O_4_ ratio. A maximum pore size increase, from 11 to 90 μm, is observed from p-TPU to TCP105. As the CF:Fe_3_O_4_ ratio increases from 1:0.5, to 1:0.75, and to 1:1 (TCP105 to TCP110), Table 2 shows that the maximum pore size progressively reduces from 90 μm to 26 μm for 1:0.5; from 30 μm to 26 μm for 1:0.75; and from 26 μm to 12 μm when the CF:Fe_3_O_4_ ratio was 1:1. When the filler contents increases from 1 wt.%, to 3 wt.% and to 7 wt.%, the general trend is to have smaller pores.

Also, it is noticeable that the fracture surface in the TCP105 sample is smoother than that of p-TPU, and as the CF:Fe_3_O_4_ ratio increases, the surfaces become rougher. In the TCP710 sample, the fracture surfaces show large dimples. This rough surface appearance is an indication of good interfacial adhesion between the p-TPU and the fillers. The increased roughness and the reduction in the pore size can be interpreted as an increase in the surface tension in the liquid composite, which hinders the polymer bloating during the vapor-induced phase separation (VIP) method, due to the filler incorporation. Li et al. [37] explained that a similar situation occurs for TPU/graphene/Fe_3_O_4_ composites; the presence of magnetite nanoparticles at the interfaces of the pores causes changes in the structure in terms of dimensions and morphology of the pores as the Fe_3_O_4_ ratio increases during the vapor-induced phase separation process. On the other hand, while a small number of carbon fibers are seen on the fractured surfaces in composites containing 1 wt.% filler (TCP105, TCP107, and TCP110), more carbon fibers are observed as the amount of the filler increases. Figure 4 inset shows the observed number of exposed fibers with respect to the CF:Fe_3_O_4_ ratio for the three filler contents. It is noticeable that as the amount of magnetite and filler content increases, the number of exposed fibers becomes more evident, which could be related to a reduced interaction between the filler and the p-TPU matrix. The non-uniform dispersion of the filler may potentially affect the material’s EMI shielding activity by limiting reflection.

**Figure 5:**
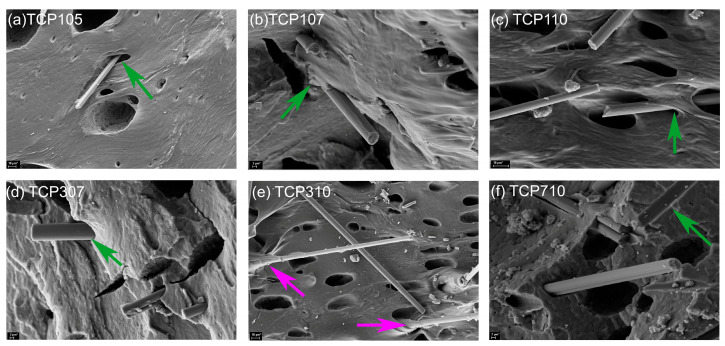
Selected SEM micrographs of the TCP composites in the regions where the interfaces between the exposed fibers and the p-TPU matrix are evident. Sample names are indicated in the micrographs.

For a more detailed visualization of the interactions between the fillers and the p-TPU matrix, Figure 5 presents selected SEM images, showing the regions where the exposed fibers are evident. In TCP105, TCP107, TCP110, and TCP307, fibers are not wet by the matrix; in the case of TCP710, the fibers are completely detached from the matrix as indicated by the green arrow, even leaving the fiber imprint where the fiber was separated from the matrix. In the TCP310 sample, the fibers are partially wrapped by the p-TPU, as indicated by purple arrows, indicating that not only the surface modification with Fe_3_O_4_ nanoparticles, but the filler loading influences the filler–matrix homogenous interaction [38]. The pores and the presence of voids between the fillers and the p-TPU, as observed from the SEM micrographs, shall modify the intrinsic impedance of the composites.

### 3.4. Thermal Analysis CF/Fe_3_O_4_/p-TPU Composites

**Figure 6:**
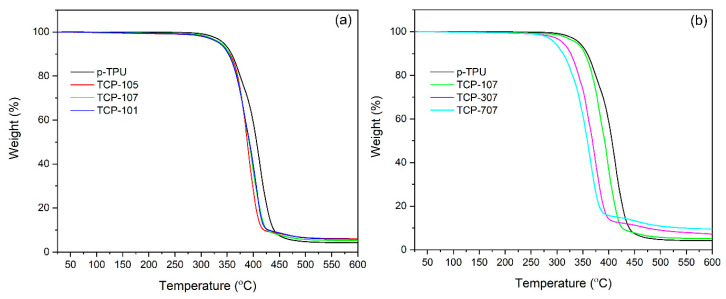
TGA curves of neat p-TPU and p-TPU composites: (**a**) varying Fe_3_O_4_ contents in the 1 wt.% filler (samples TCP105, TCP107, and TCP110); (**b**) varying the filler contents with 7 wt.% filler (samples TCP107, TCP307, and TCP707).

Thermogravimetric analysis (TGA) was performed to investigate the effects of CF/Fe_3_O_4_ filler on the thermal stability of the composites, through the percentage of weight loss versus temperature curves under nitrogen atmosphere for the neat TPU and its composites, as shown in Figure 6. Figure 6a shows the effects of changing the CF:Fe_3_O_4_ ratio (1:0.5, 1:0.75, 1:1) on thermal stability in neat TPU and TPU composites containing 1 wt.% CF/Fe_3_O_4_. Figure 6b compares thermal stability in TPU composites depending on the 1, 3, and 7 wt.% amount of CF/Fe_3_O_4_ filler materials for a constant CF:Fe_3_O_4_ (1:0.75) ratio. In all the cases, p-TPU decomposes at a slightly higher temperature than the composites and produces the lowest char yield. When the CF:Fe_3_O_4_ ratio increases from 0.5 to 1, a slight decrease in the onset and the decomposition temperatures is observed, but with respect to the filler ratio, the change is around 5 °C. Also, the char yield is similar, as expected from the same filler contents in the composite. On the other hand, when the filler contents increased from 1 wt.% to 7 wt.% at a fixed CF:Fe_3_O_4_ ratio of 1:0.75, the onset and the decomposition temperatures have a larger variation, as well as the onset and decomposition temperature, and the char yield.

Table 3 shows the data obtained from the TGA curve, including the onset degradation temperature at a 2% weight loss (T_onset_), the temperature corresponding to 50 wt.% weight loss values (T_50_%), and the temperatures of the maximum weight loss (T_max_), as well as the char yield values at 600 °C of the neat p-TPU and CF/Fe_3_O_4_/p-TPU composites. Neat TPU and CF/Fe_3_O_4_/p-TPU composites display identical thermal degradation behavior, which can be separated into two main stages. The first stage occurs at temperatures between 280 and 350 °C and is attributed to the cleavage of the urethane linkage in the TPU hard segment, involving polyol and isocyanate. The second stage occurs at temperatures between 370 and 480 °C and is attributed to the cleavage of polyol and diisocyanate into smaller molecules in the TPU soft segment [39]. The highest weight loss at 600 °C was obtained for the neat p-TPU sample (95.84%). The maximum weight loss value decreases with the addition of CF/Fe_3_O_4_, ranging between 94.84 and 90.45% compared to neat p-TPU. The maximum decomposition rate temperature (T_max_), i.e., the temperature at which the weight loss rate reaches its maximum, decreases from 446 °C to 425–391 °C with the addition of CF/Fe_3_O_4_. The decrease in degradation temperature with the addition of CF/Fe_3_O_4_ compared to neat p-TPU can be explained by the fact that the fillers act as nucleation sites for the degradation reaction, leading to a lower activation energy and degradation temperature [8,40]. Although the high heat absorption capacity of CF is expected to contribute to the increase in degradation temperature [41], like our previous study on TPU/GO/PPy/Fe_3_O_4_ composites, the decrease in the maximum degradation temperature of TPU is due to the presence of nano Fe_3_O_4_ attached to the CF acting as a depolymerization catalyst [8,42]. The initial decomposition temperatures of p-TPU composites varied between 302 °C and 309 °C, depending on the CF/Fe_3_O_4_ filler ratio, such as 1:0.5 and 1:0.75, and the initial decomposition temperature decreased by 3.4% compared to p-TPU. In addition, the onset temperature of p-TPU composites decreases from 303 °C to 282 °C when the CF/Fe_3_O_4_ filler ratio increases from 1 wt.% to 7 wt.%. In addition, it is worth noting that the char yields obtained for CF/Fe_3_O_4_/p-TPU composites at 600 °C vary between 9.55% and 5.16% compared to the 4.16% char yield obtained for neat p-TPU. TCP707 composite containing 7 wt.% CF/Fe_3_O_4_ filler with a CF:Fe_3_O_4_ ratio of 1:0.75, showed a 130% increase in char residue levels compared to the neat p-TPU, due to the increase in iron residue.

### 3.5. Dielectric Properties and Shielding Efficiency

**Figure 7:**
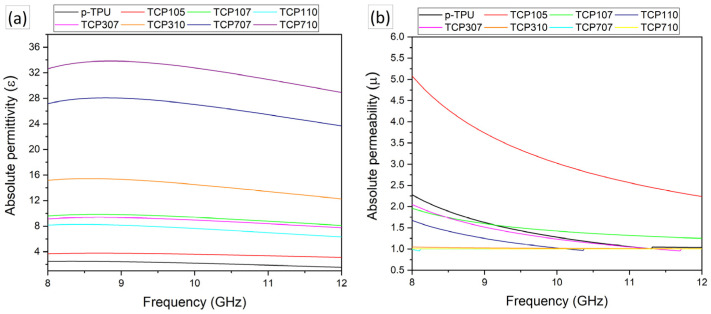
Frequency-dependent (**a**) relative permittivity (ε) and (**b**) permeability (μ) values for p-TPU and CF/Fe_3_O_4_/p-TPU composites.

As the electromagnetic shielding capabilities of composite materials depend on their dielectric properties, such as permittivity and permeability, the values of these properties in their compounds were investigated. In this study, absolute permittivity values were used to compare composite samples that had significant differences in their microstructure (e.g., porosity, conductive additive content, and morphological changes). Absolute permittivity describes a material’s ability to become polarized when exposed to an electric field. This enables the dielectric behavior of the material to be described without normalization to free space permittivity. Figure 7a presents the absolute permittivity (ε) of pristine TPU (p-TPU) and several TPU-based composite formulations (TCP105, TCP107, TCP110, TCP301, TCP307, TCP710, TCP707) measured in the X-band frequency range of 8–12 GHz. The results reveal distinct differences in dielectric behavior depending on the composition and microstructure of each sample. All samples exhibit dielectric stability between 8 and 12 GHz, signaling that the materials maintain predictable and frequency-independent dielectric behavior in the X-band region. The absolute permittivity (ε) values presented in Figure 7a reveal that p-TPU exhibits low ε values and remains constant around ε ≈ 1.5–2.4, highlighting the poor electrical polarization capacity and energy storage of the unfilled, porous polymer. The TCP105 composite, the sample with the lowest CF to Fe_3_O_4_ ratio, exhibited a permittivity value of ε ≈ 3 just above TPU, which is related to the low polarization resulting from the interface interaction between the CF/Fe_3_O_4_ filler and the matrix. As can be seen from the dielectric permittivity results, the dielectric permittivity values gradually increase with the loading of the CF/Fe_3_O_4_ filler and the increase in the CF/Fe_3_O_4_ ratio in the filler material. This enhancement is most likely due to the combined effects of electrically conductive carbon fibers [43] and polarizable Fe_3_O_4_ nanoparticles, which significantly alter the dielectric response of the polymer matrix. TCP710 exhibits the highest permittivity, stabilizing around ε ≈ 28–33, and TCP710 shows the second highest permittivity values, ε ≈ 23–28. The slight downward trends observed in TCP710 and TCP707 composites imply a slight decrease in permittivity with increasing frequency. This indicates that the dipole and interfacial polarization mechanisms are only weakly sensitive to the field frequency in this range.

Conversely, Figure 7b shows the absolute permeability, µ, for neat p-TPU and p-TPU composites with varying CF/Fe_3_O_4_ loadings. The TCP105 specimen exhibits a significantly enhanced magnetic response (μ > 1) within the 8–12 GHz frequency range. In contrast, the TCP310, TCP707, and TCP710 composites exhibit transmission values converging to a single value (μ ~ 1), while TCP110 and TCP307 show a curve decreasing with frequency and approaching μ ~ 1 after 10 GHz. This behavior may initially appear contradictory given the higher Fe_3_O_4_ loading in TCP110, TCP307, TCP310, TCP707, and TCP710 composites. This can be attributed to the suppression of magnetic loss mechanism by the conductive network enabled by the CF network and the limitations imposed by the frequency of magnetic resonance in the X band [44]. This suggests that Fe_3_O_4_ is primarily involved in improving electromagnetic impedance matching, rather than acting as a dominant loss contributor.

**Figure 8:**
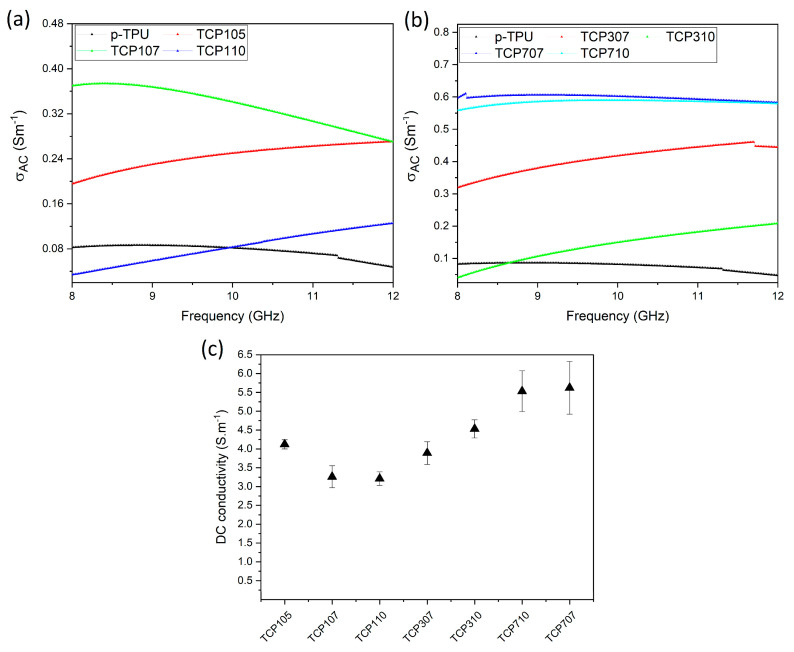
AC electrical conductivity of (**a**) and (**b**) neat p-TPU and p-TPU composites containing different weight ratios of CF/Fe_3_O_4_ and their (**c**) DC conductivity values with standard deviations.

The alternating current (AC) electrical conductivity (σ_ac_) of neat p-TPU and CF/Fe_3_O_4_/p-TPU composites was calculated with Equation (4). Figure 8a,b shows the frequency-dependent σ_ac_ conductivity values of p-TPU and CF/Fe_3_O_4_/p-TPU composites within the 8–12 GHz frequency range, highlighting the influence of filler loading and presence of porosity on electromagnetic shielding performance. Figure 8a shows that TCP107 exhibits the highest σ_ac_, which decreases slightly as the frequency increases. In contrast, TCP105 and TCP110 show a steady increase, indicating improved charge transport at higher frequencies. p-TPU demonstrates the lowest conductivity across the range. As shown in Figure 8b, the composites with higher filler loadings (TCP707 and TCP710) achieve significantly greater σ_ac_ values, which lie between 0.56 and 0.59 Sm^−1^ while remaining stable within the frequency range. In contrast, TCP307 and TCP310 display moderate conductivity values, exhibiting a gradual upward trend. The mean σ_ac_ conductivity value of the neat p-TPU sample was measured as 0.076 ± 0.011 S/m. The composites TCP105, TCP107, and TCP110 show mean σ_ac_ values of 0.244 ± 0.02 S/m, 0.335 ± 0.03 S/m, and 0.082 ± 0.02 S/m, respectively. The mean σ_ac_ values of the TCP 307, TCP 310, TCP 707, and TCP 710 composites were found to be 0.40 ± 0.03 S/m, 0.141 ± 0.04 S/m, 0.598 ± 0.007 S/m, and 0.583 ± 0.007 S/m, respectively, which are higher than those of the neat p-TPU. Figure 8c shows the variation of DC conductivity values of p-TPU and CF/Fe_3_O_4_/p-TPU composites with the amount of CF/Fe_3_O_4_ filler and CF:Fe_3_O_4_ ratio. It should be noted that the DC conductivity of pure TPU could not be measured because the volume resistance of TPU is considerably higher than the measurement limits of the four-point conductivity device. However, the literature provides a DC electrical conductivity value of 10^−11^ S/m for the TPU polymer [45]. A noteworthy point in Figure 8 is that the DC conductivity of the composites is nearly 10 times greater than their AC conductivity values. The fact that DC conductivity exceeds AC conductivity indicates that a continuous conductive network is formed via the carbon fibers and Fe_3_O_4_ clusters within the TPU, enabling dominant ohmic electron transport. As with AC conductivity values, the DC conductivity values of TCP710 and TCP707 composites, which contain a high proportion of filler material, are better than those of other composites. In Figure 8, the TCP105 sample exhibits superior electrical conductivity values for both AC and DC compared to TPU composites containing 1% CF/Fe_3_O_4_. This is likely due to the microstructural advantages of the TCP105 composite. Specifically, TCP105 exhibits a unique combination of low overall porosity and uniformly distributed ~1.9 micrometer pores (Table 1). These pores maintain a continuous flow path for DC transmission, while supporting a strong interfacial capacitive microstructure. This enhances both DC and alternating current (AC) conductivity, while suppressing effective permeability (Figure 7a). This increases the internal impedance compared to other composites. Similarly, the DC electrical conductivity values of TPU composite filled with 5–20 wt.% carbon nanofiber and fabricated via solution mixing method [46] were observed to be comparable to those obtained in the present work.

**Figure 9:**
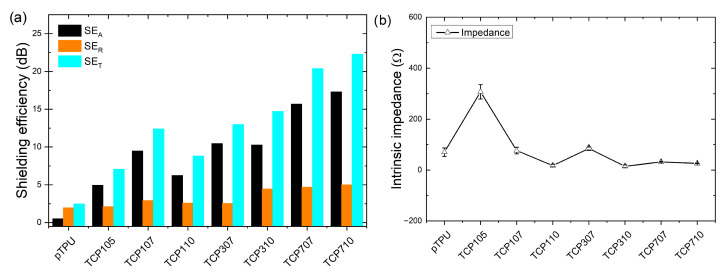
(**a**) Average SE_A_, SE_R_, and SE_T_ values and (**b**) average intrinsic impedance of the composites. Since the standard deviations in Figure (**a**) have very small values ranging from 1.27 × 10^−14^ to 4.8 × 10^−15^, they are not clearly visible on the graph.

In this study, the electromagnetic interference (EMI) shielding effectiveness and electrical properties of CF/Fe_3_O_4_ hybrid-filled thermoplastic porous polyurethane (p-TPU) composites were investigated in the X-band frequency range through various parameters, including absorption (SE_A_), reflection (SE_R_), total shielding effectiveness (SE_T_), and electrical conductivity (σ), the later discussed in the previous section. Figure 9a illustrates the average values of absorption (SE_A_), reflection (SE_R_), and total shielding effectiveness (SE_T_) calculated from Equations (5)–(10), and in Figure 9b, the intrinsic impedance calculated with Equation (11), for neat p-TPU and CF/Fe_3_O_4_/p-TPU composite samples is shown. The total shielding efficiency (SE_T_) curves and the absorption (SE_A_) and reflection (SE_R_) contributions along the X-band, as well as the impedance vs. frequency curves, are shown in Figure S5. Figure 9a shows that neat p-TPU exhibits negligible electromagnetic wave absorption due to the lack of magnetic permeability and electrical conductivity, but shows an SE_T_ of about 2.47 dB, which could be attributed to the porous morphology, which causes multiple internal scattering [47]. However, p-TPU SE_A_ values remain close to 0 dB, indicating its low effectiveness as an EMI shielding material. Among the composites, TCP105 demonstrates the lowest SE_T_ value with 7.05 dB, as well as absorption performance in the range of 4.9 dB. This limited performance stems from its low filler content, which hinders electric-related reflection and magnetic absorption, and the continuous conductive network is not yet fully formed. Thus, the shielding mechanism is dominated by dielectric loss mechanisms due to charges accumulating at filler–matrix interfaces in this sample [47]. Correspondingly, the maximum SE_T_ value of 22.28 dB was obtained in the sample containing 7 wt.% CF/Fe_3_O_4_ with CF:Fe_3_O_4_ ratio of 1:1. An increasing trend in SE_T_ values is observed in CF/Fe_3_O_4_/p-TPU composites as both the percentage of CF/Fe_3_O_4_ filler material and the CF:Fe_3_O_4_ ratio increase. This behavior can be attributed to the formation of a well-developed conductive network within the TPU matrix. This significantly enhances the dielectric loss (ε″), as shown in Figure S6, and may also result in magnetic loss due to the presence of Fe_3_O_4_. Although the high DC conductivity (Figure 8) suggests the presence of a conductive network formation and an electromagnetic response dominated by conductivity, the superior absorption efficiency compared to reflection can be explained by considering the porous microstructure of the TPU-based composites. The presence of micro- and meso-scale pores in TPU composites (Figure S4 and Table 1) introduces several additional mechanisms that reduce impedance mismatch [48] and increase internal attenuation, causing the material to efficiently absorb incoming electromagnetic waves rather than reflect them. In order to characterize the absorption efficiency of TPU composites as being due to magnetic loss, impedance mismatch caused by internal voids and interfacial polarization, skin depth calculations were performed on composite samples using DC electrical conductivity values at a frequency of 10 GHz. For TPU-based composites with a sample thickness of 3 mm, skin depth values were found to vary between 1.5 mm and 2.8 mm. Skin depth values are comparable to sample thicknesses, but are found to be slightly smaller than sample thicknesses. This places the TPU-based composites in an appropriate absorption regime, where electromagnetic waves can sufficiently penetrate the bulk structure and minimize reflection-induced responses.

As depicted in Figure 9, the increase in the proportions of CF and Fe_3_O_4_ significantly enhances EMI shielding efficiency, particularly by boosting the absorption (SE_A_). Despite the high CF:Fe_3_O_4_ ratio (1:1) in the TCP110 composite, its abnormally low SE_A_ and SE_T_ values are most likely due to the lowest porosity (9.38%) of this composite [47], as seen in Table 1. Upon examination of p-TPU composites, it is observed that as the filler content increases, the absorption (SE_A_) values show a noticeable and consistent rise, while the reflection (SE_R_) values exhibit limited variation, remaining relatively stable. This finding emphasizes that absorption is the dominant mechanism for EMI shielding (confirmed by skin depth values in Table 4), as reflected by the continuous increase in SE_A_ values, although the pore size effect in the absence of the magnetic filler deserves to be studied in future work.

With respect to the intrinsic impedance, Figure 9b exhibits a non-monotonic dependence on the CF/Fe_3_O_4_ filler loading. The pronounced impedance maximum is observed for TCP105 composite due to its limited charge transport and relatively low permittivity (Figure 7a), arising from insufficient conductive network formation. TCP307 composite also shows a slightly elevated average intrinsic impedance. Unlike TCP105, this situation is likely related to a temporary balance between impedance matching and dielectric loss. The gradual increase in permittivity (Figure 7a) and electrical conductivity (Figure 8) associated with interfacial polarization stabilizes average intrinsic impedance values of TCP310, TCP707, and TCP710 composites at relatively low values. The average impedance values show little variation at high filler loading (TCP707 and TCP710), but these composites maintain higher impedance levels (see Figure S5) compared to those with lower CF/Fe_3_O_4_ filler loading. This behavior correlates well with the observed EMI shielding performances of the composites. At high CF/Fe_3_O_4_ loadings, the combination of average impedance stabilization and relatively high impedance levels creates a greater internal loss due to microstructural heterogeneity and the dominant loss mechanism. This enables efficient electromagnetic wave entry and results in highly effective, absorption-dominant electromagnetic interference (EMI) shielding.

**Figure 10:**
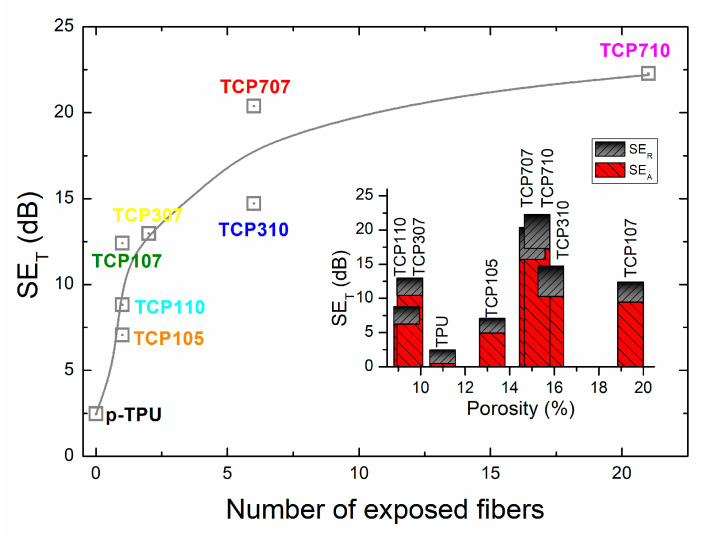
Total shielding response vs. the number of exposed fibers from the SEM images in Figure 4 and total shielding response disaggregated in SE_R_ and SE_A_ contributions vs. porosity of the composites (inserted figure).

The dispersion of EM waves is further increased by the presence of filler material separated from the p-TPU matrix within the porous structure. Figure 10 presents the relation of the total shielding efficiency (SE_T_) vs. the number of exposed fibers in the fracture surfaces observed in the SEM micrographs, as a measure of the fiber detachment from the p-TPU matrix. The inset shows the relationship between the total shielding efficiency, disaggregated by the SE_A_ and SE_R_ contributions, and the calculated porosity, to investigate the possible influence of texture on absorption due to multi-scattering within pores and shielding due to magnetite/filler contents, as previously discussed.

Figure 10 shows the SE_T_ increment with the number of exposed fibers. The lowest SE_T_ is p-TPU, for which the only shielding contribution (2.47 dB) is the porosity (ca. 11%). The first section of curve in Figure 10 comprises the SE_T_ increase related with increasing magnetite contents of the samples with 1 wt.% filler, where the second lowest sample is TCP105 with the lowest magnetite load and highest conductivity; in this sample, the contribution to SE_T_ is partially from reflection due to scattering within the pores as shown in Figure 10 inset and some absorption (SE_A_) due to magnetite load. In Figure 10 inset is noticed that samples with 1 wt.% of filler load (TCP107 and TCP110) have similar SE_R_ while the SE_A_ response is not linear with the CF:Fe_3_O_4_ ratio as discussed before (TCP105 < TCP110 < TCP107), indicating than TCP107 sample which has the larger porosity, may owe its enhanced SE_A_ to the increased air–solid interfaces [49]. Correspondingly, Figure 10 shows a further non-linear increase of SE_T_ with the number of exposed fibers, in the sequence TCP307 < TCP310 < TCP707 < TCP710. Analyzing the SE_R_ and SE_A_ contributions to SE_T_ as depicted in Figure 10 inset, for these samples, SE_R_ variation is related to the porosity, while SE_A_ increase is related to the filler load. Thus, the behavior of the incrementally loaded filler loading implies a higher magnetite content, as well as increased micro voids between the decorated fibers and the matrix, i.e., the interfacial interactions between the filler and the matrix and multiple internal reflections, have an important influence on the EMI shielding properties of the composites [50].

**Figure 11:**
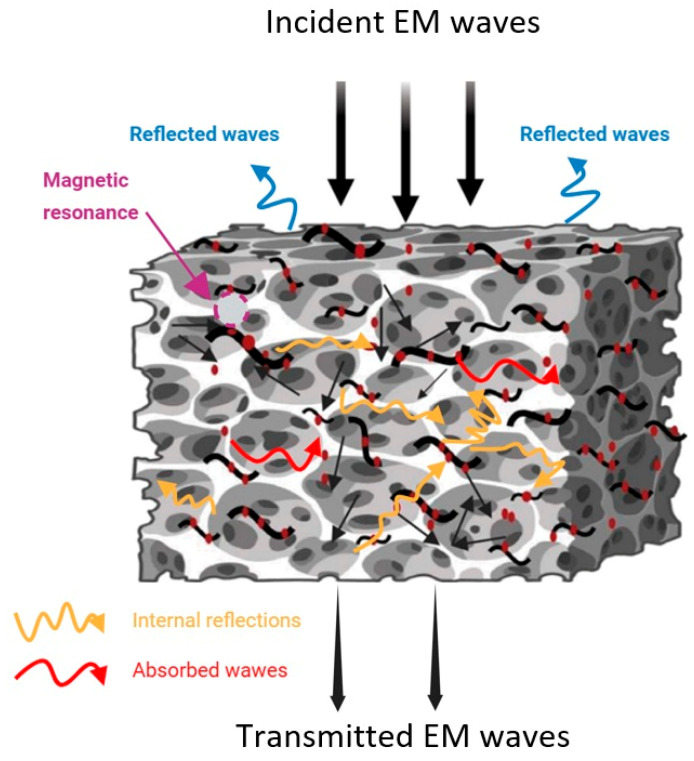
EMI shielding mechanisms of CF/Fe_3_O_4_/p-TPU composites (The red dots represent Fe_3_O_4_ nanoparticles, and the black wavy lines represent carbon fibers).

Figure 11 proposes a scheme of the scattering process of incoming electromagnetic waves in CF/Fe_3_O_4_/p-TPU composites and the possible shielding mechanisms of the produced composites against electromagnetic waves. When incident EM waves encounter the composite, some EM waves are reflected due to impedance mismatch with the porous structures, while most of the waves are attenuated by absorption due to magnetic loss, and the rest by interfacial reflections in the conductive fibers and within the porous structure of the CF/Fe_3_O_4_/p-TPU composite. As seen from the SEM images taken from the fractured surfaces, the porous structure in the composites promotes multiple scattering, which consumes EM waves. The porous surfaces improve the impedance matching for air entry, greatly reducing reflection at the air–absorber interfaces [5]. The closed pores in CF/Fe_3_O_4_/p-TPU polymer composites facilitate EM wave reflection and attenuation. EM waves entering the material are dissipated due to repeated reflections at the porous structure interfaces due to impedance mismatch, which would be enhanced with filler that is internally detached from the p-TPU matrix. Fe_3_O_4_ in CF/Fe_3_O_4_ embedded in TPU contributes to magnetic loss and absorption efficiency, while the presence of carbon fibers promotes the polarization loss of electromagnetic waves.

To compare the performance of the composites prepared in this work with some reported in the literature, Table 5 presents the main characteristics of the reported materials, which are related to the performance, such as the matrix and filler type, the filler loading, and density and thickness for shielding efficiency. The total shielding attenuation at the X-band frequency range is used as a figure of merit with which to compare respective performances.

## 4. Conclusions

This study investigated the EMI shielding performance and dielectric properties of porous thermoplastic polyurethane (p-TPU) composites reinforced with CF/Fe_3_O_4_ hybrid fillers with different weight ratios and CF:Fe_3_O_4_ ratios. The findings demonstrate the synergistic effect of conductive carbon fibers (CFs) and magnetic Fe_3_O_4_ particles (which contributes to the impedance matching and microstructural heterogeneity) with the porous structure in enhancing the shielding efficiency of TPU composites across the X-band frequency range (8–12 GHz). In general, the increased loading of CF/Fe_3_O_4_ fillers in porous TPU matrix promoted the formation of CF-CF conductive networks, and enhanced interfacial polarization, thereby increasing the electrical conductivity and, consequently, the EMI shielding performances of the composites. Among the studied composites, the TCP710 sample containing 7 wt.% CF/Fe_3_O_4_ filler with a CF/Fe_3_O_4_ (1:1) ratio exhibited superior total EMI shielding efficiency (SE_T_), achieving a maximum of approximately 22.28 dB, with significant contributions from absorption (SE_A_ 17.3 dB). This performance can be attributed to the optimized CF/Fe_3_O_4_ ratio (1:1) with balanced impedance matching air–TPU interface, which reduces EM wave at the surface while a larger fraction of EM waves can penetrate into interior of the composites, where they are dissipated through multiple loss mechanism (conductive losses due to CFs, interfacial polarization at CF/Fe_3_O_4_ interfaces, and multiple reflections due to porous structures) leading to enhanced SE_A_. Furthermore, the fact that the skin depth values of the composites are slightly lower than their thicknesses ensures that TPU-based composites are absorption-dominant materials that allow EM waves to penetrate them. Magnetic permeability analysis and the VSM characterization confirmed that soft magnetic Fe_3_O_4_ nanoparticles contribute to magnetic loss, further supporting absorption-dominant shielding in TCP707 and TCP710. Morphological analysis using SEM revealed the effects of the filler contents and the CF:Fe_3_O_4_ ratio in the distribution of fillers within the TPU matrix, which proved to be critical for effective interfacial polarization and energy dissipation.

In summary, this study demonstrates that CF/Fe_3_O_4_/p-TPU composites can achieve high-performance EMI shielding through complementary reflection and absorption mechanisms with a strong influence of interfacial filler–matrix interactions and pore size distribution, for applications requiring lightweight, flexible, and effective EMI shielding, particularly in high-frequency environments.

## Figures and Tables

**Table 1:** Summary of the prepared CF/Fe_3_O_4_/pTPU composites, with density and porosity data.

Sample	Filler wt.%	CF wt.%	Fe_3_O_4_ wt.%	Density g/cm^3^	Std Dev.	Porosity %
pTPU	0	0	0	1.077	0.022	10.99
TCP105	1	1	0.5	1.101	0.051	13.22
TCP107	1	1	0.75	1.0541	0.176	19.43
TCP110	1	1	1	0.9792	0.153	9.38
TCP307	3	1	0.75	1.059	0.027	9.52
TCP310	3	1	1	1.060	0.032	15.85
TCP707	7	1	0.75	1.0325	0.0403	15.02
TCP710	7	1	1	1.1095	0.047	15.23

**Table 2:** Average and maximum pore sizes of the prepared CF/Fe_3_O_4_/p-TPU composites.

Sample	Average Pore Size (µm)	Std Deviation	Maximum Pore Size (µm)
pTPU	2.7	0.9	11
TCP105	1.9	0.5	90
TCP107	4.0	1.7	40
TCP110	3.0	1.0	26
TCP307	3.0	2.0	30
TCP310	2.2	0.7	26
TCP707	3.0	1.0	26
TCP710	2.0	1.0	12

**Table 3:** Thermal analysis data of p-TPU and CF/Fe_3_O_4_/p-TPU composites.

Sample Name	T_onset_ (°C)	T_50%_ (°C)	T_max_ (°C)	Max Weight Loss (%)	Char Residue (%) @T_600_ °C
p-TPU	320	406	446	95.84	4.16
TCP105	302	388	420	93.98	6.02
TCP107	309	392	425	94.84	5.16
TCP110	303	391	424	94.22	5.78
TCP307	295	370	402	92.81	7.19
TCP707	282	359	391	90.45	9.55

**Table 4:** Calculated skin depth (δ) values at 10GHz as a function of real relative permeability (µ_r_) and DC electrical conductivity for TPU-based composites.

Sample Name	µ_r_ (Real)	σ_DC_ (Sm^−1^)	Skin Depth (δ)
p-TPU	-	-	-
TCP105	2.55	4.12	1.55
TCP107	1	3.26	2.79
TCP110	1.009	3.21	2.80
TCP307	1.22	3.89	2.31
TCP310	1	4.53	2.36
TCP707	1	5.62	2.12
TCP710	1	5.53	2.14

**Table 5:** Overview of materials employed for EMI shielding (NA is “not applicable”).

Composite Foam	Matrix	Filler Type	Filler Loading	Thickness (mm)	Density (g/cm^3^)	EMI SE_T_ (dB)	Ref.
TPU/RGO	TPU	RGO	6.5 wt.%	1.8	1.1	21.8	[51]
PS/RGO	PS	RGO	30 wt.%	2.5	NA	29	[52]
PU/graphene	PU	graphene	10 wt.%	6	0.03	39.4	[53]
PP/CF	PP	CF	10 vol.%	3.2	NA	24.9	[54]
PEI/graphene@Fe_3_O_4_	PEI	Graphene/Fe_3_O_4_	10 wt.%	2.5	0.4	14.3	[55]
pTPU/CF:Fe_3_O_4_	TPU	CF/Fe_3_O_4_	7 wt.%	3	1.1	22.28	This work

## Data Availability

The original contributions presented in this study are included in the article. Further inquiries can be directed to the corresponding author.

## Supplementary Materials

The following supporting information can be downloaded at: https://www.mdpi.com/article/10.3390/polym18010019/s1, Figure S1: Calibrating distance in SEM image; Figure S2: Image segmentation; Figure S3: Measurement configuration and pore size analysis; Figure S4: Average pore sizes calculated from the SEM images of the prepared composites; Figure S5: Electromagnetic shielding curves of the prepared composites (SEA: absorption, SER: reflection, SET: total) (a) p-TPU, (b) TCP105, (c) TCP107, (d) TCP110, (e) TCP307, (f) TCP310, (g) TCP707, (h) TCP710; (i) Impedance vs frequency curves for all the samples; Figure S6. Variation of the imaginary permittivity (ε″) as a function of frequency for p-TPU, and TPU-based composites measured in the X-band region.
