# Supplementary material for: Investigating the Structural, Thermal, Electric, Dielectric, and EMI Shielding Properties of Porous Thermoplastic Polyurethane Reinforced with Carbon Fiber/Magnetite Fillers
Source: Polymers (Basel). 2025 Dec 21;18(1):19. doi: 10.3390/polym18010019 (PMC12787563; doi:10.3390/polym18010019)
Supplement: Supplementary file 1 [file polymers-18-00019-s001.zip › polymers-3993723-supplementary.pdf]

Supplementary Information

Investigating the structural, thermal, electric, dielectric, and EMI shielding properties of porous thermoplastic polyurethane reinforced with carbon fiber/magnetite fillers

Hülya Kaftelen-Odabaşı[1*], Ümmühan Kaya[2], Akın Odabaşı[3], Selçuk Helhel[4], Fernando Ruiz-Perez[5], Felipe Caballero-Briones[5]

[1]School of Civil Aviation, Dept. of Aircraft Maintenance and Repair, Fırat University, 23200 Elazığ, Turkey; hkodabasi@firat.edu.tr

[2]Graduate School of Natural and Applied Science, (Aerospace Science &Technologies), Fırat University, 23200 Elazığ, Turkey; ummhan.kayaa@gmail.com

[3]Engineering Faculty, Dept. of Metallurgical and Materials Engineering, Fırat University, 23200 Elazığ, Turkey; odabasia@firat.edu.tr

[4]Engineering Faculty, Dept. of Electrical & Electronics Engineering, Akdeniz University, 07058 Antalya, Tur-key; selcukhelhel@akdeniz.edu.tr

[5]Instituto Politécnico Nacional, Materiales y Tecnologías para Energía, Salud y Medio Ambiente (GESMAT), CICATA Altamira, 89600 Altamira, Mexico; fcaballero@ipn.mx

*Correspondence: hkodabasi@firat.edu.tr

Calculation of pore size distribution

ImageJ was the primary software used to determine the pore size distribution of the samples through the analysis of SEM images. ImageJ is an open-source image-processing tool developed at the National Institutes of Health and the Laboratory for Optical and Computational Instrumentation, which enables the analysis and processing of various types of images, including the generation of histograms, line profile plots, angle measurements, and particle and pore size distributions.

The procedure for determining the pore size distribution was conducted as follows:

1. **Calibration of pixel-to-length ratio.**

The pixel-to-unit scale was established using the *Straight* tool to draw a line along the scale bar of the SEM image. Then, the option *Analyze* > *Set Scale* was selected, and the known distance and corresponding units of the scale bar were entered.

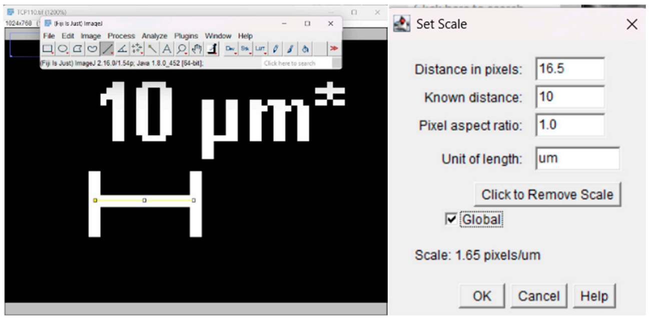

Figure S1. Calibrating distance in SEM image

2. **Image segmentation using automatic global thresholding.**

The image was segmented using an automatic global thresholding technique to highlight the features of interest (particles or pores) from the background. This was done through *Image > Adjust > Threshold*. The highlighted regions correspond to the pores. After applying the threshold settings, the image was converted into a binary (black-and-white) representation. *(Reference: Schneider, C., Rasband, W., & Eliceiri, K. NIH Image to ImageJ: 25 years of image analysis. Nat Methods 9, 671–675 (2012) https://doi.org/10.1038/nmeth.2089 .*

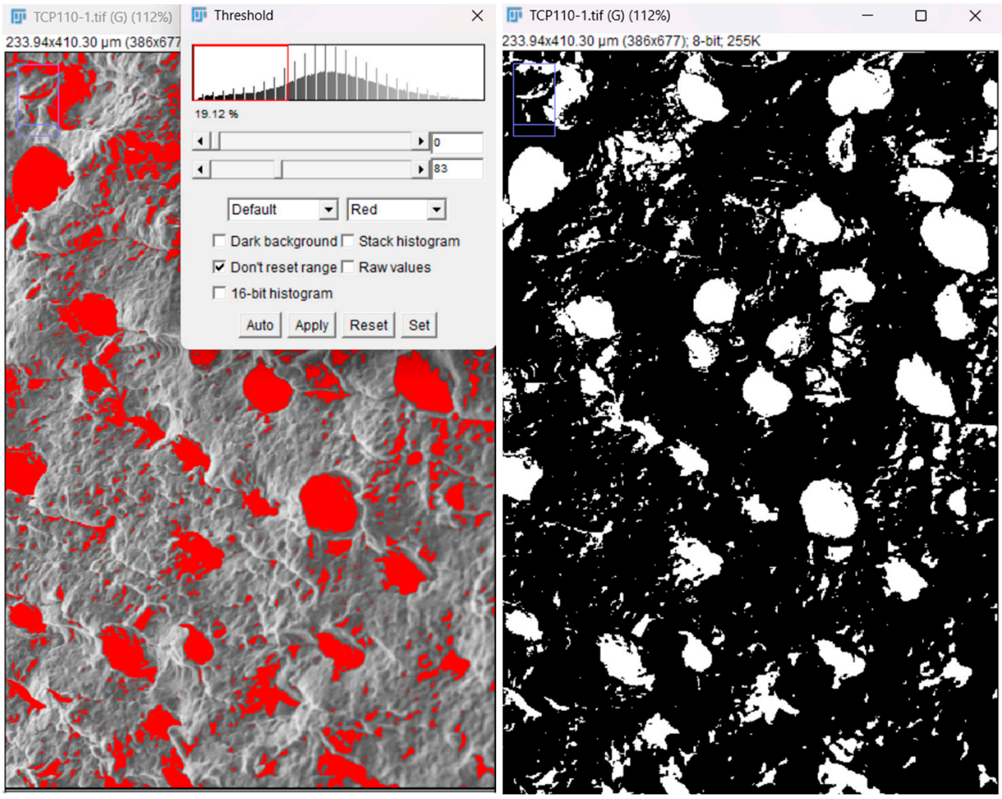

Figure S2. Image segmentation

3. **Measurement configuration and pore-size analysis.**

Measurement parameters were set through *Analyze > Set Measurements*. Finally, pore measurements were obtained using *Analyze > Analyze Particles*, which generated a table containing the measured values of the analyzed regions. These values were subsequently used to construct the pore size distribution.

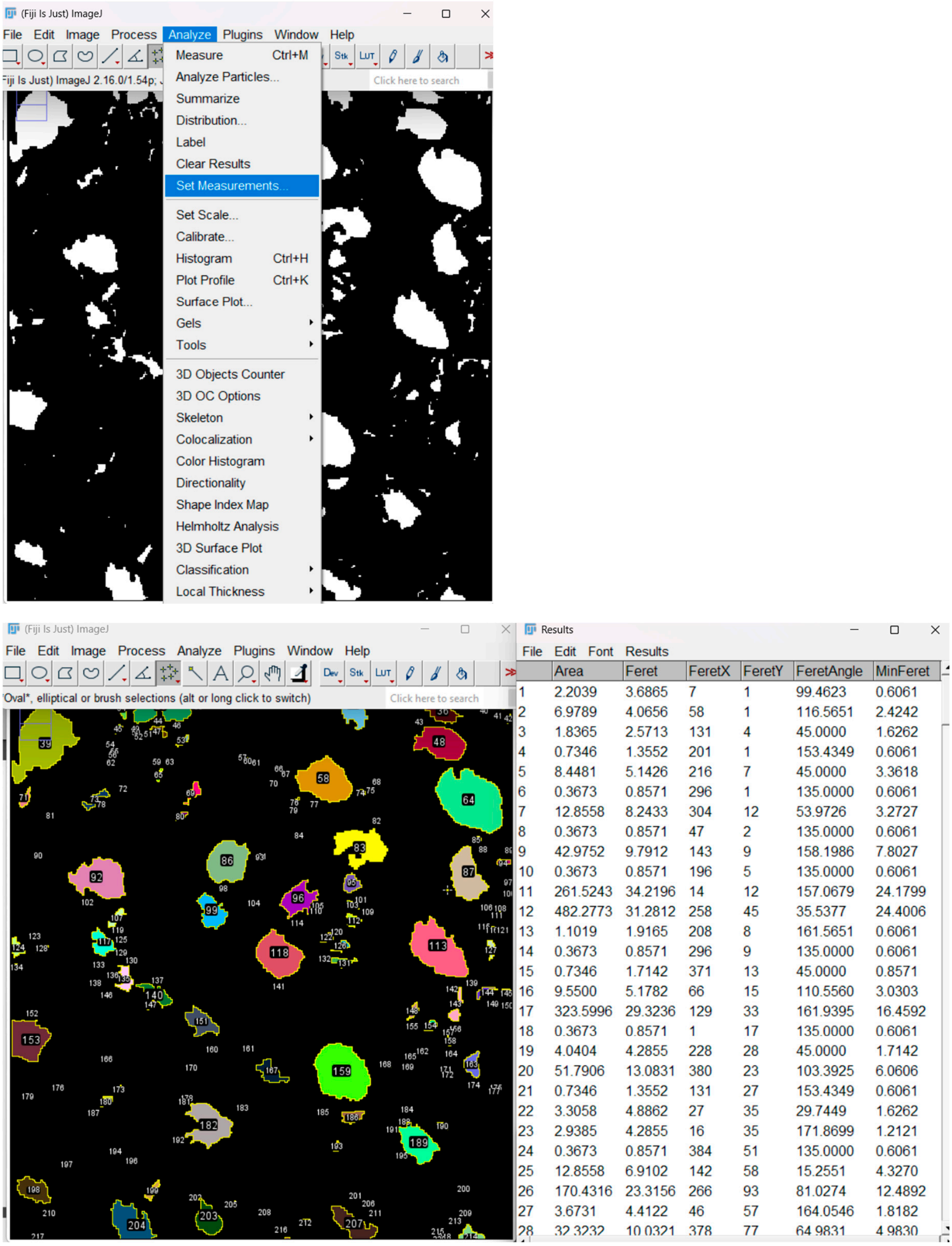

Figure S3. Measurement configuration and pore size analysis

Figure S4 presents the pore size distribution of the p-TPU and the TPU/CF/Fe_3O_4 composites described in Table 1 of the manuscript, obtained from the SEM micrographs.

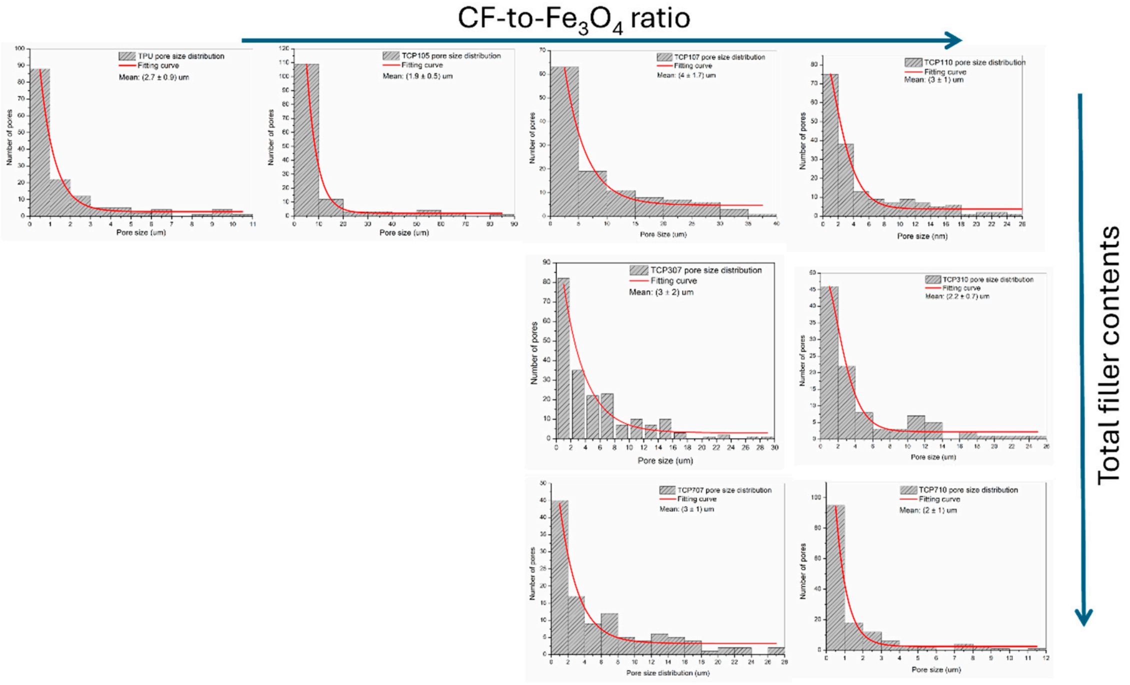

Figure S4. Average pore sizes calculated from the SEM images of the prepared composites.

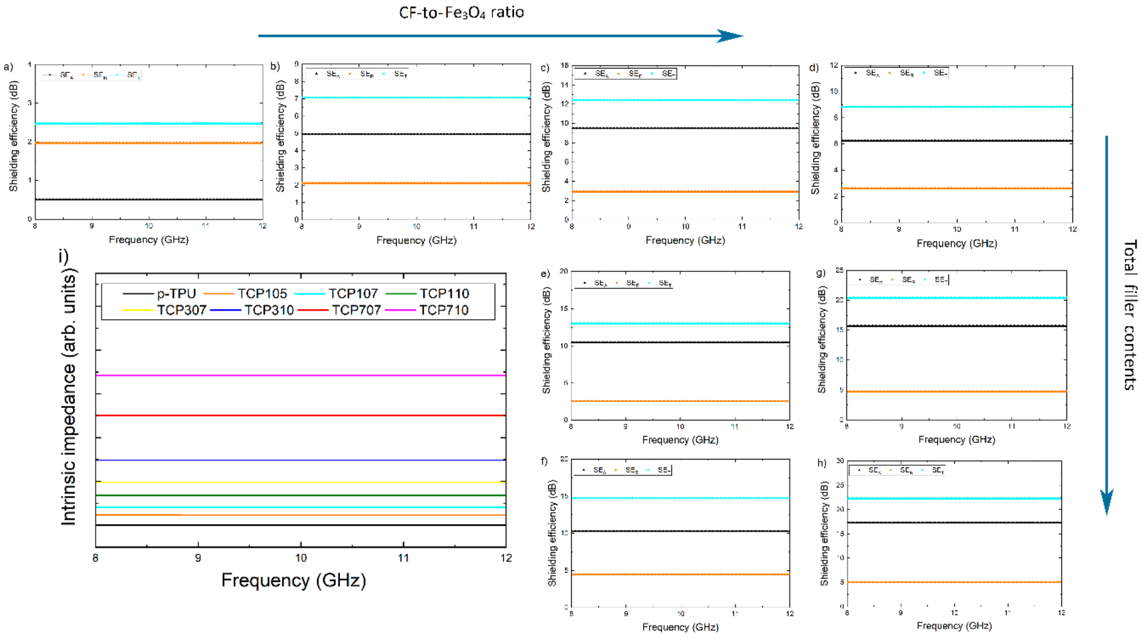

Figure S5. Electromagnetic shielding curves of the prepared composites (SE_A: absorption, SE_R: reflection, SE_T: total) a) p-TPU, b) TCP105, c) TCP107, d) TCP110, e) TCP307, f) TCP310, g) TCP707, h) TCP710; i) Impedance vs frequency curves for all the samples

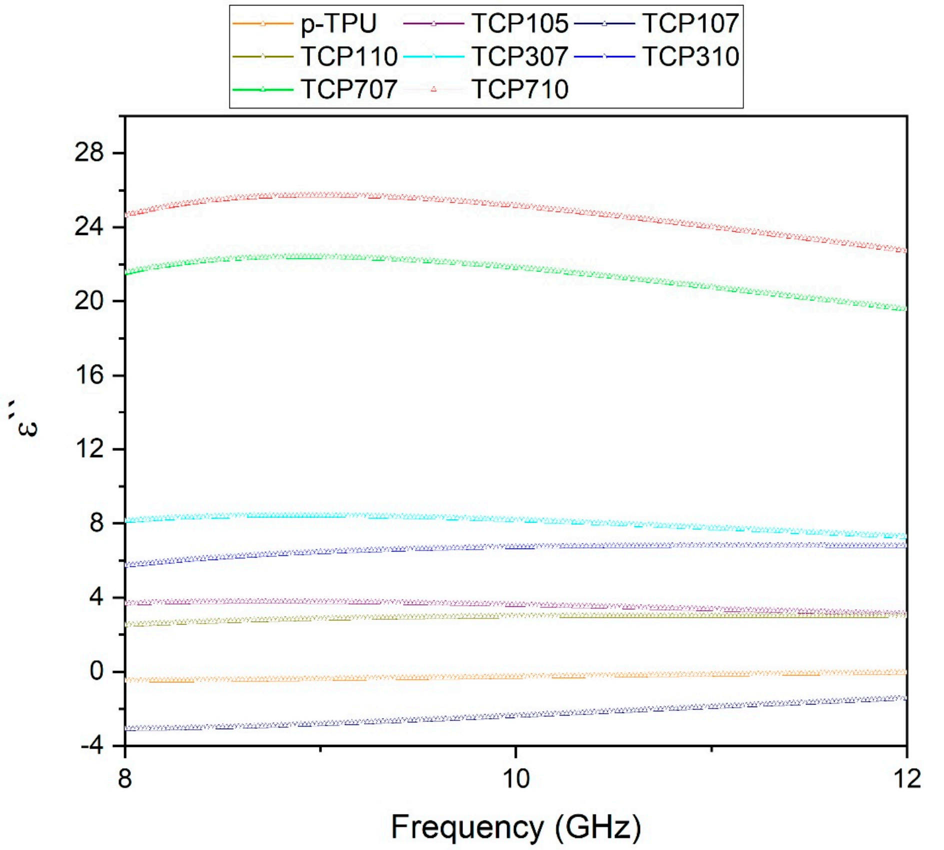

Figure S6. Variation of the imaginary permittivity (ε") as a function of frequency for p-TPU, and TPU-based composites measured in the X-band region.
